# Extracts of *Physalis peruviana* Protect Astrocytic Cells Under Oxidative Stress With Rotenone

**DOI:** 10.3389/fchem.2018.00276

**Published:** 2018-07-20

**Authors:** Natalia Areiza-Mazo, Jorge Robles, Jairo A. Zamudio-Rodriguez, Lisandro Giraldez, Valentina Echeverria, Biviana Barrera-Bailon, Gjumrakch Aliev, Amirhossein Sahebkar, Ghulam Md Ashraf, George E. Barreto

**Affiliations:** ^1^Departamento de Nutrición y Bioquímica, Facultad de Ciencias, Pontificia Universidad Javeriana, Bogotá, Colombia; ^2^Departamento de Química, Facultad de Ciencias, Pontificia Universidad Javeriana, Bogotá, Colombia; ^3^Departamento de Química e Exatas, Universidade Estadual do Sudoeste da Bahia, Jequié, Brazil; ^4^Facultad de Ciencias de la Salud, Universidad San Sebastián, Concepción, Chile; ^5^Bay Pines VA Healthcare System, Research and Development, Bay Pines, FL, United States; ^6^Institute of Physiologically Active Compounds, Russian Academy of Sciences, Chernogolovka, Russia; ^7^GALLY International Biomedical Research Consulting LLC., San Antonio, TX, United States; ^8^School of Health Science and Healthcare Administration, University of Atlanta, Johns Creek, GA, United States; ^9^Neurogenic Inflammation Research Center, Mashhad University of Medical Sciences, Mashhad, Iran; ^10^Biotechnology Research Center, Pharmaceutical Technology Institute, Mashhad University of Medical Sciences, Mashhad, Iran; ^11^School of Pharmacy, Mashhad University of Medical Sciences, Mashhad, Iran; ^12^King Fahd Medical Research Center, King Abdulaziz University, Jeddah, Saudi Arabia

**Keywords:** *Physalis peruviana*, uchuva, phenolics, astrocytes, oxidative stress, rotenone

## Abstract

The use of medicinal plants to counteract the oxidative damage in neurodegenerative diseases has steadily increased over the last few years. However, the rationale for using these natural compounds and their therapeutic benefit are not well explored. In this study, we evaluated the effect of different *Physalis peruviana* extracts on astrocytic cells (T98G) subjected to oxidative damage induced by rotenone. Extracts of fresh and dehydrated fruits of the plant with different polarities were prepared and tested *in vitro*. Our results demonstrated that the ethanolic extract of fresh fruits (EF) and acetone-dehydrated fruit extract (AD) increased cell viability, reduced the formation of reactive oxygen species (ROS) and preserved mitochondrial membrane potential. In contrast, we observed a significant reduction in mitochondrial mass when rotenone-treated cells were co-treated with EF and AD. These effects were accompanied by a reduction in the percentage of cells with fragmented/condensed nuclei and increased expression of endogenous antioxidant defense survival proteins such as ERK1/2. In conclusion, our findings suggest that ethanolic and acetone extracts from *P. peruviana* are potential medicinal plant extracts to overcome oxidative damage induced by neurotoxic compounds.

## Introduction

Free radicals and reactive oxygen species (ROS) are common by-products of cellular metabolism and xenobiotic exposure (Circu and Aw, 2010). These molecules play important roles in the defense against pathogens and mediate intracellular communication and regulation (Schieber and Chandel, 2014). They also interfere with highly complex biological pathways that regulate cell growth, cell death and senescence. This interference is exerted through various mechanisms such as acting on phagolysosome formation and enzymatic degradation, autophagy, chemoattraction, inflammation, and acting as redox messengers (Paiva and Bozza, 2014). However, excessive ROS generation can lead to cell damage through destabilization of the mitochondrial oxidative phosphorylation supercomplexes, which in turn will stimulate further ROS production. This vicious cycle in most cases can induce oxidative stress and subsequent damage to vital biomolecules such as lipids, proteins, and DNA (Halliwell, 2007; Chaban et al., 2014). Overproduction of ROS has been associated with several chronic diseases such as cancer, autoimmune disorders, cardiovascular disease, and neurodegenerative diseases (Halliwell, 2007; Kim et al., 2010; Blach-Olszewska et al., 2015; Gasiorowski et al., 2017).

Brain tissue is especially vulnerable to oxidative stress owing to its high oxygen consumption and the oxidizing capacity of monoamine oxidase on neurotransmitters such as dopamine that results in H_2_O_2_ overproduction (Barreto G. E. et al., 2011). Other factors such as the presence of highly reactive copper and iron ions, high concentrations of polyunsaturated fatty acids and low concentrations of the antioxidant enzymes catalase and glutathione peroxidase account for the suceptibility of the brain tissue to oxidative damage (Cui et al., 2004; Albarracin et al., 2012). Oxidative stress has been associated with mitochondrial dysfunction, a pathological mechanism that occurs in most neurodegenerative diseases including Alzheimer's disease (AD) and Parkinson's disease (PD) (Cabezas et al., 2012, 2015; Denzer et al., 2016). In this regard, defects in mitochondrial energy metabolism may result in a decrease in high-energy phosphate reserves, antioxidant defense impairment, deterioration of membrane potential, and deregulation of calcium homeostasis, thereby leading to excitotoxicity and neuronal death (Federico et al., 2012; Chaturvedi and Flint Beal, 2013; Abeti and Abramov, 2015). Therapeutic efforts aiming at improving brain antioxidant defense are essential to alleviate damage especially during neurodegeneration (Barreto et al., 2017). To counteract neuronal degeneration, astrocytes, the second most abundant cells in the brain after neurons (Barreto G. E. et al., 2011; Barreto G. et al., 2011; Garzon et al., 2016), play a vital role. This protective role of astrocytes against oxidative damage is exerted through releasing endogenous antioxidant species such as glutathione and superoxide dismutase (SOD), or removing glutamate (Barreto G. et al., 2011).

There is an increasing interest in studying the neuroprotective effects of natural products and functional foods. Phytochemicals are exogenous antioxidants that are widely present in fruits and vegetables. For example, polyphenols from berries (blueberries and grapes) have shown antioxidant and anti-aging effects (Kim et al., 2010; Vuong et al., 2010; Xia et al., 2010; Bornsek et al., 2012; Queiroz et al., 2017), suggesting potential neuroprotective actions. Additionally, previous animal studies have shown that consumption of flavonoid-rich foods improves cognitive function, vascular function and synaptic plasticity in the brain (Rendeiro et al., 2015).

In a continuing search for natural compounds to be used for the prevention and treatment of PD, the present study investigated the properties of different fractions isolated from the fruits of *Physalis peruviana*, commonly known as goldenberry. This plant possesses antioxidant and anti-inflammatory properties associated with its polyphenol content (Horn et al., 2015; Yildiz et al., 2015). Goldenberry has a high content of vitamins A, C, E, D, and B complex, polyphenols, withanolides, and carotenoids (Xu et al., 2017; Etzbach et al., 2018). Some of these components of goldenberry act as “scavengers” of free radicals and can prevent cell damage and neuroinflammation induced by oxidative stress (Abdel Moneim, 2016; Sang-Ngern et al., 2016). The present work aimed to perform a preliminary evaluation of the antioxidant properties of goldenberry components in PD. We assessed the effect of different fractionated extracts of P. peruviana on the response of astrocytic cells to rotenone, an inhibitor of the complex I of the mitochondrial electron transport chain that is used to generate a cellular model of PD.

## Materials and methods

### Chemicals

The chemicals used to isolate the plant extracts, including ethanol, dichloromethane, petroleum benzine, acetone, and ethyl acetate, were purchased from Merck (Merck & Co., Inc., Kenilworth, NJ, USA).

### Plant material

The fruits of *P. peruviana* plant were obtained from an organic farm located at 2,900 m above sea level in Cundinamarca, Colombia. Before use, each fruit was selected based on the absence of the signs of bacterial or fungal infection, or structural damage. The vegetal material was obtained with the calyx present to preserve the integrity of the fruit. Fruits were divided in two parts of ~1,000 g. One part was heat dehydrated at 45°C for 4 days, macerated and stored until the day of use. Another part was homogenized in a food chopper and used fresh.

### Preparation of crude extracts

The preparation followed the protocol developed previously by Domínguez (1979). Briefly, fresh fruit was submerged into an ethanol solution at room temperature (RT) under mild agitation. Then, ethanol was evaporated using a rotary evaporator (BUCHI, RE 111. Flawil, Switzerland) at 40°C until a pure ethanolic fresh fruit (EF) fraction was obtained. About 80% of the ethanolic extract was used for liquid-liquid fractionation, and the remaining 20% was used to perform bioassays. The first fraction was obtained with petroleum benzine, then dichloromethane and finally with ethyl acetate. Each fraction was evaporated to obtain the respective fractions (BF), (DF), and (AF). The final material was lyophilized (FreeZone 2.5 Liter Benchtop Freeze Dry System, Labconco©, Kansas City, MO, USA) to obtain the lyophilized extract (L). On the other side, the dehydrated fruit was submerged into a petroleum benzene solution at RT with mild agitation, then evaporated to obtain the respective fraction (Benzene Dehydrated; BD). Subsequently, the resulting residual material was extracted first with dichloromethane, then with acetone, and lastly with ethanol, and each solvent was evaporated to obtain the pure fractions dichloromethane dehydrated (DD), acetone dehydrated (AD), and ethanolic dehydrated (ED). Materials were submerged, with mild agitation, in 2 L of each solvent for a period of 2 days to obtain the individual extracts. The obtained fractions were weighed and diluted in 99.9% DMSO and stored at 20°C.

### Determination of the total phenolic content

Folin-Ciocalteu reagent (F9252. Sigma-Aldrich®, St. Louis, MO, USA) assay was used for determining the content of phenols (Mena et al., 2012). The testing mix consisted of 50 mg extracts (100 μL), 800 μL of distilled water, and 100 μL of Folin-Ciocalteau. The mix was incubated in the dark for 8 min. Subsequently, 50 μL of 7.5% sodium carbonate was added and the new mix solution incubated for 1 h. Finally, the phenolic content was determined spectrophotometrically measuring the absorbance of the mix at 760 nm and a standard curve made with known concentrations of gallic acid.

### Cell culture

T98G [T98-G] Homo sapiens brain glioblastom (ATCC® CRL-1690™) cell line was maintained under exponential growth in Eagle Modified by Dulbeco (DMEM) (12-917F Lonza® Walkersville, MD, USA) culture medium, supplemented with 10% fetal bovine serum (FBS), antibiotics (penicillin/streptomycin) and amphotericin at 37°C. Cell cultures were maintained in a humidified atmosphere containing 5% CO_2_ (Ávila Rodriguez et al., 2014).

### Drug treatments

Cells were seeded in multi-well plates and allowed to grow for 24 h. Afterwards, the cultured cells were serum-deprived for 24 h prior to treatments. Then, cultured cells were exposed to rotenone [50 μM] (R8875. Sigma-Aldrich®, St. Louis, MO, USA) for 24 h, as described by Cabezas et al. (2015).

### Cell viability

T98G cell viability was tested using MTT (5 mg/ml stock solution) [3-(4,5-dimethylthi-azol-2-yl)-2,5-diphenyltetrazolium bromide] assay (M2128. Sigma-Aldrich®, St Louis, MO, USA) (Swarnkar et al., 2012; Riss et al., 2013). Cells were seeded into 96-well plates in DMEM culture media containing 10% bovine fetal serum at a seeding density of 10,000 cells per well and allowed to grow for 24 h. Afterward, cells were serum deprived for 24 h, and finally treated with golden berries extracts at 25, 50, 100 y 200 μ g/ml for 12, 18, and 24 h. Cell viability was assessed following the treatments by adding 0.45 mg/ml per well MTT solution for 4 h at 37°C in the dark. Afterwards, formazan crystals were solubilized with dimethyl sulfoxide (DMSO; 276855.Sigma-Aldrich®, St Louis, MO, USA) and the absorbance at 490 nm was determined. Each assay was performed with a minimum of six replicate wells for each condition. The amount of released formazan, which is directly proportional to the number of live cells, was determined by optical density (OD) at 540 nm in a spectrophotometer. The values were normalized to the value of the control culture without extract added containing 0.01% DMSO, which was considered 100% survival. Rotenone-treated cells were used as the control for neurotoxicity.

### Determination of reactive oxygen species (ROS)

To measure the potential neuroprotective effect of the goldenberry extracts from superoxide (O^2−^) and oxygen peroxide (H_2_O_2_) production induced by rotenone, ROS production was evaluated by cytometry and fluorescence microscopy as described (Torrente et al., 2014). Briefly, cells were seeded at a density of 25,000 cells per well into 48-well plates in a DMEM culture medium containing 10% FBS and then subjected to 24 h of serum deprivation before treatments. Then, cells were treated with 10 μM dihydroethidium (DHE; 37291. Sigma-Aldrich®, St Louis, MO, USA) or 1 μM 20,70-dichlorofluorescein diacetate (DCFDA; 35845. Sigma-Aldrich®, St Louis, MO, USA), in the dark at 37°C for 30 min. Then cells were washed in PBS, detached with trypsin (Trypsin/EDTA 500 mg/l:200 mg/L- BE02-034E. LONZA, Walkersville, MD, USA) and analyzed for flow cytometry in a Guava EasyCyteTM cytometer (Millipore, Billerica, MA, USA). Each assay was performed with a minimum of three replicates per condition.

For DHE fluorescence imaging analysis, cells were seeded at a density of 20,000 cells per well into 48-well plates in a DMEM culture medium containing 10% FBS. On the next day, cells were co-treated with 50 μM rotenone plus a plant extract. After 24 h, cells were incubated with DHE for 30 min. Finally, cells were washed with PBS and photographed in an Olympus IX53 fluorescence microscope- ex/em 340/510 nm (OLYMPUS CORPORATION, Shinjuku, Tokyo, Japan). The images were processed with Image J software (NIH, Bethesda, MD, USA), and the mean fluorescence intensity of randomly selected cells was determined by fluorescence microscopy (Olympus IX53 microscope, ex/em 340/510 nm; OLYMPUS CORPORATION, Shinjuku, Tokyo, Japan) using a 20X objective. The number of fluorescent cells was determined in at least eight randomly selected areas (0.03 mm^2^) from each experimental group. Each experiment was performed in triplicate.

### Cytoplasmic nitric oxide concentration (NO)

Nitric oxide production was evaluated by determining nitrite levels with the Griess reagent (G4410; Sigma-Aldrich®, St Louis, MO, USA). Cells were seeded at a density of 25,000 cells per well into 48-well plates in DMEM containing 10% FBS. Then, cells were subjected to serum deprivation for 24 h and co-treatment with 50 μM rotenone plus the respective extract. Once treatments were completed, the culture medium was harvested and maintained at −80°C until used. Duplicates of 100 μL of the culture medium were added to 96-well plates and mixed with 100 μL of Griess reagent. Quantification was performed using spectrophotometry (Omega Star Fluorometer; BMG LabTech, Ortenberg, Germany) measuring absorbance at 540 nm. Each assay was performed in triplicates.

### Lipid peroxidation

Lipid peroxidation is an indicator of oxidative stress and the reactive aldehydes generated, like 4-hydroxynonenal (HNE) may well act as “second toxic messengers.” To visualize lipid peroxidation, fluorescence of HNE was measured. Cells were washed twice with PBS after rotenone plus the respective extract in co-treatment, and then fixed in 4% paraformaldehyde for 10 min. Cells were permeabilized with 1% Triton X-100, diluted in PBS with 2.5% serum, for 20 min at RT, and stored at −20°C. Then, cells were immunostained with rabbit anti-HNE antibodies (1:500; ab46545, Abcam, Cambridge, MA, USA) and analyzed using fluorescence microscopy as described in section Determination of Reactive Oxygen Species (ROS).

### Nuclear fragmentation

To visualize nuclear morphology after co-treatment with rotenone and the respective extract, cells were washed twice with PBS and then fixed in 4% paraformaldehyde for 20 min (Ávila Rodriguez et al., 2014). Cells were then stained with 2.5 mg/mL DNA dye Hoechst 33342 (14533. Sigma-Aldrich®, St Louis, MO, USA) in PBS for 15 min at RT and analyzed using fluorescence microscopy as described in section Determination of Reactive Oxygen Species (ROS). Data was expressed as the percentage of cells presenting nuclear fragmentation.

### Determination of mitochondrial membrane potential (ΔΨm)

Mitochondrial membrane potential was evaluated using tetramethyl rhodamine methyl ester (TMRM). TMRM (T5428. Sigma-Aldrich®, St Louis, MO, USA) is a cell penetrating cationic fluorescent dye sequestered by active mitochondria. After 24 h of established treatments, cells were loaded in the dark with 500 nM TMRM at 37°C for 30 min. Thereafter, cells were washed with PBS and quantified using flow cytometry in a Guava R Easy CyteTM (Millipore) cytometer. As an experimental control, we used the protonophore uncoupler carbonyl cyanide m-chlorophenylhydrazine (CCCP; Sigma-Aldrich®, St Louis, MO, USA; 10 mM) to dissipate the membrane potential and define the baseline for the analysis (Cabezas et al., 2015).

### Determination of the mitochondrial volume

Mitochondrial mass was evaluated using Nonyl Acridine Orange (NAO; A1372. Invitrogen, Waltham, MA, USA), a cell penetrating cationic fluorescent dye sequestered by active mitochondria (Cabezas et al., 2015). Cells were seeded at a density of 20,000 cells per well into 48-well plates in a DMEM culture medium containing 10% FBS and then were treated according to the experimental paradigm. After 24 h of treatment, cells were loaded with 5 μM NAO at 37°C for 30 min in the dark. Afterwards, cells were washed with PBS and mitochondrial volume was evaluated using flow cytometry in a Guava R Easy CyteTM (Millipore) cytometer.

### Mitochondrial and endoplasmic reticulum calcium concentration

Mitochondrial and endoplasmic reticulum calcium concentrations were evaluated using the Rhod-2 (R1245MP, Thermo Fisher Waltham, MA, USA) and Mag-fura-2 (M1292, Thermo Fisher Waltham, MA, USA) according to the protocol described by Avila et al. with minor modifications (Ávila Rodriguez et al., 2014). Cells were seeded at a density of 20,000 cells per well into 48-well plates in a DMEM culture medium containing 10% FBS and then were treated according to the previous experimental paradigm of 24 h of serum deprivation. After 24 h of treatments, cells were incubated with 5 μM Rhod-2 or 3 μM Mag-fura-2 in standard medium for 30 min in the incubator. Single cell fluorescence was excited at 545 nm in an Omega Star Fluorometer (BMG LabTech, Ortenberg, Germany).

### Determination of antioxidant status

The expression of antioxidant molecules and the protein concentration of superoxide dismutase (SOD, Mn-SOD) (1:1,000; PA5-30604, Thermo Fisher, Waltham, MA, USA), catalase (CAT) (1:1,000; PA5-18531, Thermo Fisher. Waltham, MA, USA) and glutathione peroxidase (GPx) (1:1,500; PA5-30593, Thermo Fisher, Waltham, MA, USA) and protein kinases ERK1 (13-8600, Thermo Fisher, Waltham, MA, USA) and ERK1/2 (13-6200, Thermo Fisher, Waltham, MA, USA) were determined using Western blotting according to the protocol described by Baez-Jurado et al. (2017). β-tubulin immunoreactivity (1:100; 2128, Cell Signaling, Danvers, MA, USA) was used for protein loading and transfer. T98G cells were lysed on ice with RIPA Lysis, and Extraction Buffer Thermo Scientific™ (89900, Thermo Fisher, Waltham, MA, USA) supplemented with Halt™ Protease Inhibitor Cocktail, EDTA-free (100X) (78430, Thermo Fisher, Waltham, MA, USA). Protein content was estimated using the Pierce™ BCA Protein Assay Kit. Equal amounts of protein were dissolved in sample buffer containing 5% β-mercaptoethanol and boiled. Then, proteins were separated by electrophoresis in SDS–PAGE, transferred onto a PVDF membrane and blocked in 5% skim milk dissolved in Tris-buffered saline containing 0.05% Tween 20 (TBS-T) at RT for 1 h. The membranes were incubated at 4°C overnight with antibodies against the desired protein. The immunoreactivity was visualized by incubating the membrane with the specific secondary antibody (IRDye® Antibodies) for 1 h and detected using Odyssey CLx Imaging System Specifications (LI-COR Biosciences). The intensity of each band was quantified using Image J software (National Institutes of Health, Bethesda, MD, USA). All data are normalized to control values on each gel.

### Statistical analysis

Data were tested for normal distribution using the Kolmogorov–Smirnov test and homogeneity of variance by Levene's test. Then, data were examined using analysis of variance, followed by Dunnet's *post-hoc* test for comparisons between controls and treatments and Tukey's *post-hoc* test for multiple comparisons between the means of treatments and time points. Data is presented as mean ± SEM values. A statistically significant difference was defined at *p* < 0.05. The results were analyzed using GraphPad Prism software version 5.00 for Windows (GraphPad Software, San Diego, CA, USA).

## Results

### Cytotoxic effect of *P. peruviana* extracts on T98G cells

The analysis of the cytotoxic effect of the fruit extracts from *P. peruviana* on T98G cells allowed us to establish both the extract concentration with the least toxic effect and the optimal treatment conditions. Our results showed a significant decrease in cell viability (>50%, *p* <0.0001) in cells exposed to both petroleum benzene and dichloromethane extracts of the fresh fruit (BF, DF) as well as petroleum benzene and dichloromethane extracts of dehydrated fruit (BD, DD) at various incubation times (12, 18, and 24 h) and concentrations (25, 50, 100, and 200 μ g/ml; Supplementary Figure 1). Reductions of 78.7 or 40% in cell viability were observed when cells were exposed to 200 μg/mL of BD for 18 h (Supplementary Figure 1A) or 200 μg/Ml of DD extract for 12 h (Supplementary Figure 1B). This cytotoxic effect was independent of the concentration of the extract used and the duration of incubation with BD, DD, and BF extracts (Supplementary Figures 1A–C). DF extract showed a cytotoxic effect on T98G cells that was dependent on both duration and concentration of exposure. No reduction in cell viability was observed at 25 μg/mL DF (*p* = 0.53) for 12 h or at 25 μg/mL (*p* = 0.95) and 50 μg/mL (*p* = 0.9262) for 18 h but a cytotoxic effect was observed in all concentrations after 24 h (Supplementary Figure 1D). In contrast, ethanolic extracts (EF from fresh fruit and ED from dehydrated fruit), ethyl acetate extracts (AF), acetone extracts (AD), and lyophilized samples (L) did not affect cell viability at 25, 50, and 100 μg/mL concentrations in all three times tested (Supplementary Figures 2A–E). Indeed, some extracts such as AD (50 μg/mL for 12 h) increased cell viability by 53% (Supplementary Figure 2B). The ED (200 μ g/ml for 24 h) and EF (for 12 and 18 h) significantly decreased cell viability by 49, 30, and 43.5%, respectively (*p* = 0.0003) (Supplementary Figures 2A,C). Similarly, AD extract at the higher concentration (200 μg/mL for 18 and 24 h) had a toxic effect, decreasing cell viability by 74.7 and 71.9%, respectively (*p* < 0.0001) (Supplementary Figure 2B).

### EF extract increased cell survival against rotenone in T98G cells

**Figure 1:**
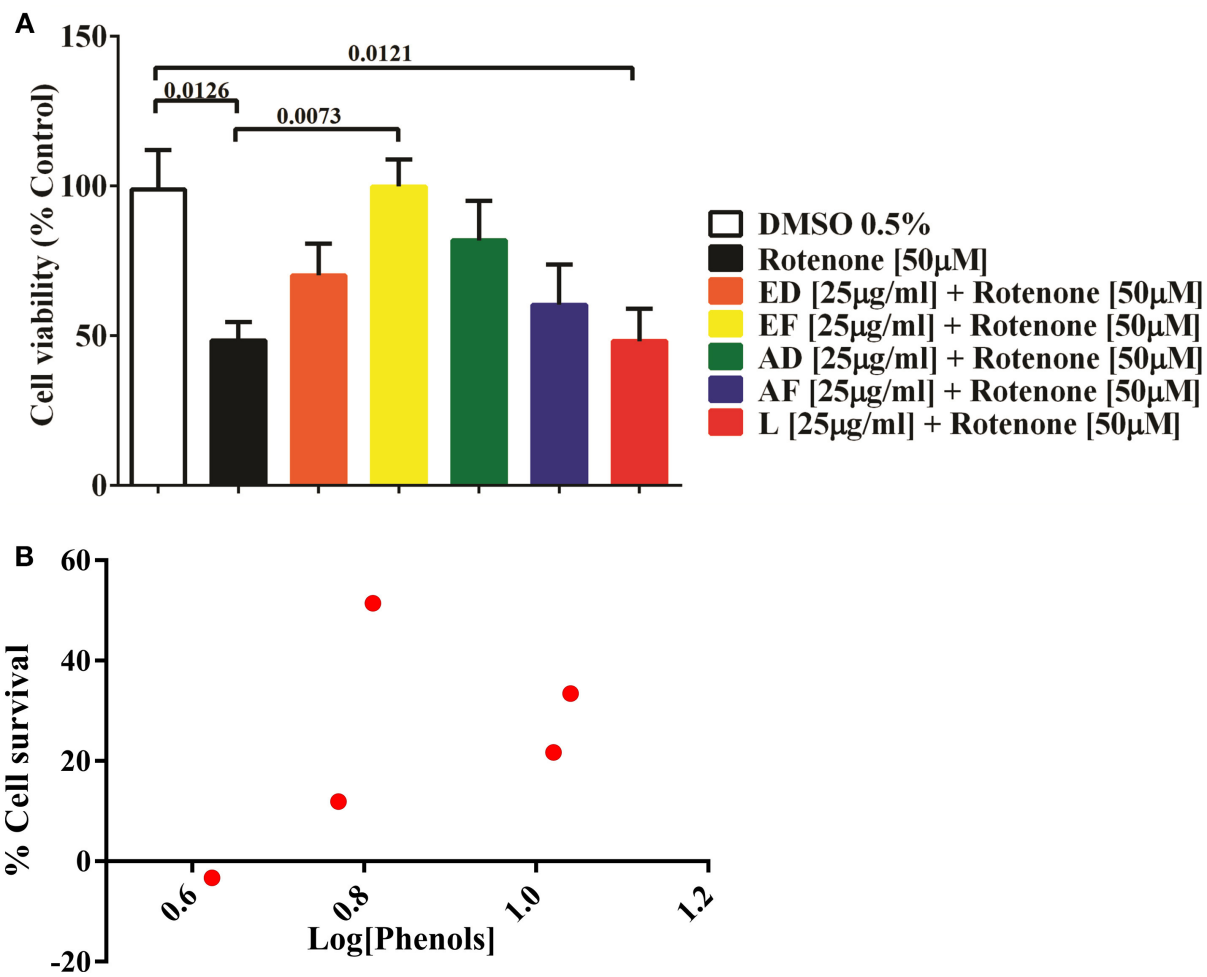
Effect of extracts of *Physalis peruviana* on the viability of T98G cell. **(A)** Percentage of survival of T98G cells measured by MTT against the toxic effect of 50μM rotenone for 24 h and simultaneous treated with extracts EF, ED, AF, AD, and L (25μ g/ml). A 52% decrease in cell survival (*p* = 0.0126) was detected after exposure to rotenone. In the co-treatment with EF extract, cellular survival was increased in 51% (*p* = 0.073), followed by AD extract in 33% (*p* = 0.1290), ED in 21.7% (*p* = 0.4839), AF in 11.9% (*p* = 0.8990). There was no significant effect of the lyophilized extract. **(B)** Correlation of the phenol content and percentage of cell survival of the extracts EF, ED, AF, AD, and L before the insult with 50μM rotenone. The content of phenols in the extracts EF, ED, AF, AD, and L was determined by the Folin-Ciocalteu method.

Based on the toxicity assays, ED, EF, AD, AF, and L extracts at 25 μg/mL were selected to assess their cytoprotective effects. Cells were co-treated with the plant extracts and 50 μM rotenone for 24 h. The results showed that rotenone reduced cell viability by 52% (*p* = 0.013). However, EF extracts improved cell survival by 51% (*p* = 0.0073). Also, a non-significant increase (*p* < 0.05) in cell viability was attained with AD (33.4%) and ED (21.7%), AF (11.9 %), while L extract diminished viability by 3% (Figure 1A).

#### Correlation of phenol content of fruit extracts and protective effect on T98G cells

Quantification of phenols in extracts ED, EF, AD, AF, and L was performed. The highest content of phenolic compounds was present in the AD extract with 11.13 mg gallic acid equivalents/g of dry mass, followed by the ED extract with 10.6 mg equivalents (Table 1). The lowest concentrations were found in the EF extract with 6.5 mg equivalents, while AF presented 5.9 mg equivalents and the freeze-dried extract with 4.2 mg gallic acid equivalents/g sample (Table 1). No correlation was found between quantity of phenols and percentage of survival (Figure 1B).

**Table 1:** Quantification of phenols and relation of percentage of survival of T98g cells in relation with Rotenone control treatment.

**Extract**	**mg gallic acid equivalents/grams of dry mass**	**% Cell survival**
Dehydrated fruit ethanolic extract (ED)	10.6	21.7
Fresh fruit ethanol extract (EF)	6.5	51.4
Dehydrated fruit acetone extract (AD)	11.1	33.4
Dehydrated fruit ethyl acetate extract (AF)	5.9	11.9
Freeze- dried (L)	4.2	−3.31

### EF and AD extracts reduced ROS levels in cells upon oxidative damage

**Figure 2:**
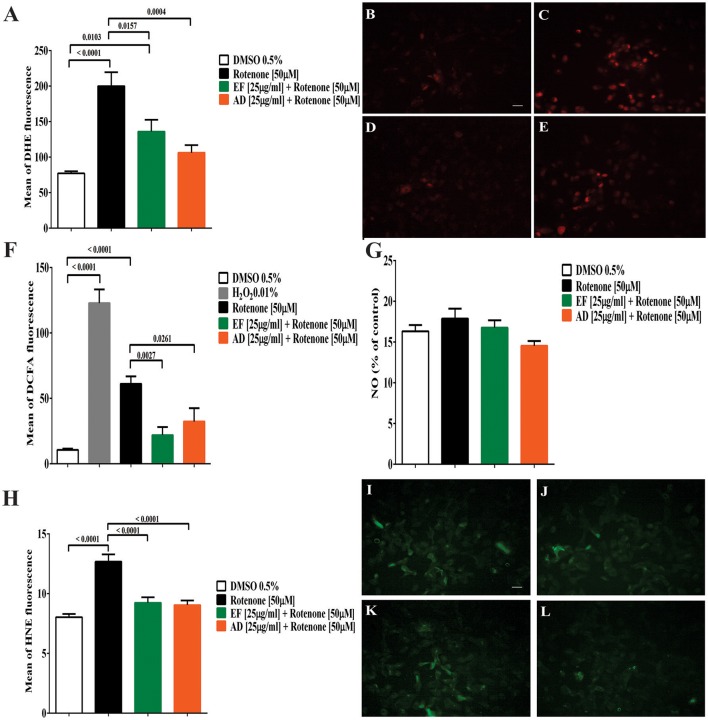
Production of reactive oxygen and nitrogen species (ROS) lipid peroxidation in T98G cells. **(A)** Mean fluorescence of DHE from superoxide radical in T98G cells. Quantification of superoxide radical in T98G cells after co-treatment with 50 μM rotenone plus EF or AD at 25 μ g/ml by flow cytometry. Determination of DHE by fluorescence microscopy. **(B)** Cells exposed to 0.5% DMSO; **(C)** Cells exposed to 50 μM rotenone (*p* < 0.0001); **(D)** Cells exposed to 50 μM rotenone plus 25 μ g/ml EF (*p* = 0.0027); **(E)** Cells exposed to 50 μM rotenone plus 25 μ g/ml AD (*p* = 0.0261). **(F)** Mean fluorescence of DCFA in T98G cells. Quantification by flow cytometry of hydrogen peroxide in T98G cell cultures after co-treatment with 50 μM rotenone plus EF and AD (25 μ g/ml). **(G)** Nitrite concentration in T98G cells determined by Griess reagent after treatment with 50 μM rotenone and co-treatment with EF and AD at 25 μ g/ml. **(H)** Mean fluorescence of HNE in cell cultures of T98G after co-treatment with rotenone 50 μM plus EF and AD at 25 μ g/ml. Determination of HNE by fluorescence microscopy. **(I)** Cells exposed to 0.5% DMSO; **(J)** Cells treated with 50 μM rotenone (*p* < 0.0001); **(K)** Cells co-treated with 50 μM rotenone plus EF (25 μ g/ml; *p* < 0.0001); **(L)** cells co-treated with 50 μM rotenone and 25 μ g/ml AD (*p* < 0.0001). Bar scales: 10 μm.

The formation of superoxide radical and hydrogen peroxide showed a significant increase by 160% (*p* < 0.0001) for superoxide and over 200% (*p* < 0.0001) for hydrogen peroxide in cells exposed to rotenone (Figures 2A,C,F). Similarly, DCFA fluorescence increased from 10.7 to 60 in rotenone-treated cells and decreased to 22 and 32.3 (*p* = 0.0027 and *p* = 0.0261) in cultures co-treated with EF and AD (25 μg/mL for 24 h), respectively (Figure 2F). The formation of DHE showed a significant decrease of 82.9% (*p* = 0.016) and 121.5% (*p* = 0.0004) in cells co-treated with the extracts EF and AD at 25 μg/mL for 24 h, respectively (Figures 2A,D,E). Confirming the previous results, the fluorescence analysis indicated a higher emission of the fluorochrome DHE in cells treated with rotenone and a decrease in the cells co-treated with the EF and AD extracts (Figures 2B–E).

Analysis of the conditioned media of T98G cells co-treated with rotenone, and EF or AD extracts using the Griess reagent did not show statistically significant differences in nitrite production between the groups (Figure 2G). The lowest mean nitrite concentration was 14 ± 2 μM for AD treatment and the maximum concentration was 17 ± 5 μM when cells were treated with rotenone alone.

### Extracts of *P. peruviana* decreased lipid peroxidation in rotenone-treated cells

Peroxidation of membrane lipids was quantified as a measure of oxidative damage. Our results demonstrated that rotenone increased HNE fluorescence from 8 to 12.6. In contrast, there was a significant decrease in peroxidation levels, expressed as lower HNE fluorescence from 2.06 to 9 and 9.2 when cells were exposed to AD and EF extracts (25 μg/mL) (*p* < 0.0001), respectively (Figures 2H–L).

### EF and AD extracts preserved mitochondrial functions

**Figure 3:**
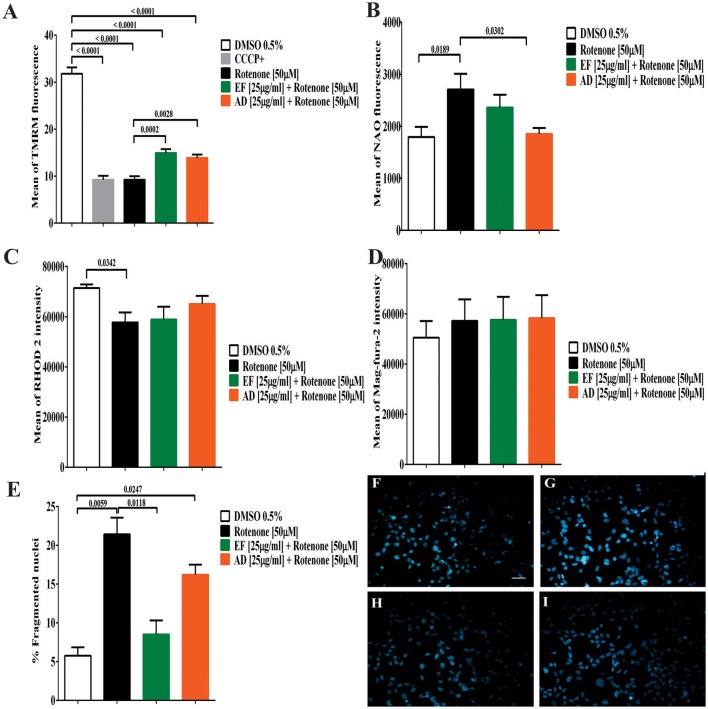
Determination of mitochondrial parameters, the content of cellular calcium and cell morphology. **(A)** Mean TMRM fluorescence in T98G cells. Determination of mitochondrial membrane potential by flow cytometry in T98G cell cultures after co-treatment with 50 μM rotenone plus EF and AD at 25 μ g/ml. **(B)** Mean fluorescence of NAO in T98G cells by flow cytometry in T98G cell cultures after co-treatment with 50 μM rotenone plus EF and AD at 25 μ g/ml. **(C)** Determination of mitochondrial calcium concentration in T98G cells. Mean fluorescence of RHOD 2, mitochondrial calcium indicator, in T98G cell cultures after co-treatment with 50 μM rotenone plus EF and AD at 25 μ g/ml. **(D)** Determination of endoplasmic calcium concentration in T98G cells. Mean fluorescence of Mag-fura-2 fluorometer, calcium indicator in cytoplasmic reticulum in cell cultures of T98G after co-treatment with 50 μM rotenone plus EF or AD at 25μ g/ml. **(E)** Percentage of cells with fragmented/condensed nuclei in T98G cells, measured by Hoescht staining after treatment with 50 μM rotenone and co-treatment with extracts EF and AD at 25 μ g/ml. Determination of Hoescht by fluorescence microscopy in **(F)** Cells exposed to 0.5% DMSO, **(G)** Cells damaged with 50 μM rotenone (*p* = 0.0059), **(H)** Cells co-treated with 50 μM rotenone plus EF 25 μ g/ml, **(I)** Cells co-treated with 50 μM rotenone and 25 μ g/ml AD (*p* = 0.0247). Bar scales: 10 μm.

Mitochondrial membrane potential (ΔΨm) showed a significant decrease of 70.98% (*p* < 0.0001) in cells exposed to rotenone (Figure 3A). In contrast, EF and AD improved mitochondrial potential by 17.9% (*p* = 0.0013) and 12.2% (*p* = 0.0097) compared to rotenone alone (Figure 3A). On the other hand, mitochondrial mass increased significantly by 51% in rotenone-insulted cells (*p* = 0.0189) and decreased by 47.6% (*p* = 0.03) after treatment with AD (25 μg/mL). Moreover, EF (25 μ g/ml) induced a 19.4% decrease in mitochondrial mass, though this difference was not statistically significant (*p* = 0.56; Figure 3B).

### Plant extracts modulate calcium levels in cells stimulated with rotenone

When calcium levels were analyzed, a significant decrease in mitochondrial Ca^2+^ levels (19%) (*p* = 0.034) was found in cells exposed to rotenone (Figure 3C). A similar decrease was observed in cells co-treated with rotenone and EF (17.5%; *p* = 0.057). However, this decrease was lower in cells treated with AD extracts (8.9%; *p* = 0.48). In contrast, ER calcium in T98G cells showed a slight, but non-significant, increase of 13% in cells stimulated with rotenone and cells co-treated with rotenone and EF or AD (25 μg/mL) relative to the control culture exposed to culture medium containing 0.05% DMSO (Figure 3D). There were no difference in ER calcium between the insulted cells and those treated with the extracts mentioned above.

### EF extract decreased nuclear fragmentation induced by rotenone

The number of fragmented and condensed nuclei quantified by fluorescence microscopy indicated an increase of 15.6% after treatment with 50 μM rotenone (Figure 3E). In parallel, a non-significant decrease in the condensed nuclei was observed in the cells co-treated with the AD extracts (5%), and a significant decrease (*p* = 0.0118) in those co-treated with EF (12.9%; Figures 3F–I).

### EF and AD extracts stimulated the expression of antioxidant enzymes

**Figure 4:**
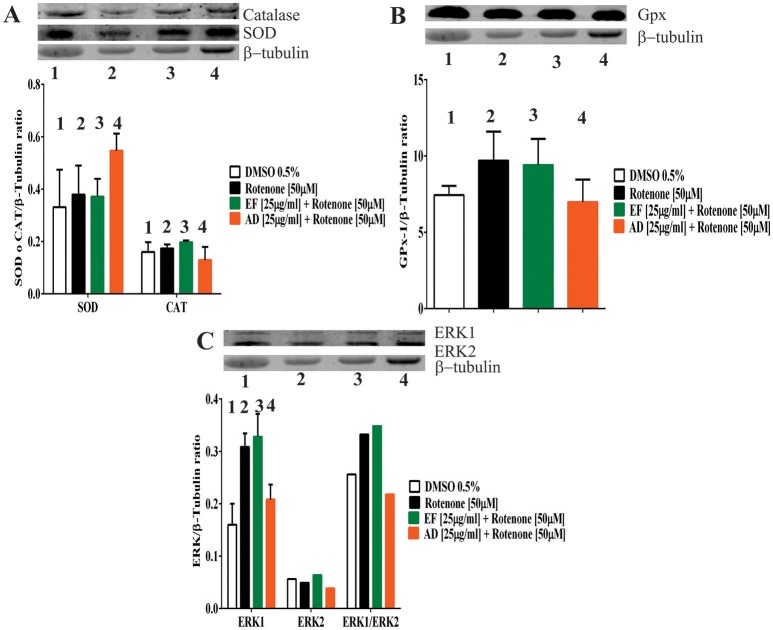
Expression of antioxidant defense and ERK proteins in T98G cells. **(A)** Expression of Superoxide dismutase (SOD) and Catalase determined by Western Blot in T98G cell cultures after co-treatment with 50 μM rotenone plus EF and AD at 25 μ g/ml. **(B)** Expression of Glutathione determined by Western Blot in T98G cell cultures after co-treatment with 50 μM rotenone plus EF and AD at 25 μ g/ml. **(C)** Expression of ERK and MAPK proteins determined by Western blotting on T98G cells after co-treatment with rotenone plus EF and AD at 25 μ g/ml.

To assess changes in the antioxidant protein levels in astrocytic cells exposed to rotenone alone or rotenone plus extracts, the expression of the antioxidant enzymes SOD, catalase and glutathione peroxidase was examined. The results showed an increase of 65% in SOD when the cells were exposed to rotenone + AD extracts in comparison to control values (Figure 4A). On the contrary, catalase expression level did not change either in cells treated with rotenone alone, rotenone + EF, or rotenone + AD extracts (Figure 4A). As for glutathione, an increase in the expression by 30.3% was observed in rotenone-treated cells, and by 26% in the rotenone + EF group compared to control. There were no significant changes in the expression of antioxidant enzymes in cells treated with AD extracts when compared to controls (Figure 4B).

ERK1 and ERK2 expression levels were also assessed. Increases by 93, 105, and 30% in ERK1 expression were observed in cells treated with rotenone (50 μM) alone, co-treated with rotenone and EF, and co-treated with rotenone and AD, respectively (Figure 4C). As for the ERK1/ERK2 ratio, there was a 29.6% increase in the rotenone-treated cells, while a 36% increase was observed after co-treatment with EF. Finally, we found a 15% reduction in ERK1/ERK2 when cells were co-treated with AD.

## Discussion

The interest in studying natural polyphenols as neuroprotective (Vauzour et al., 2008; Akinrinmade et al., 2017) and nootropic compounds has grown in recent years. A major limitation challenging the effectiveness of polyphenols for the mentioned purposes is the ability of these compounds to cross the blood-brain barrier (Subash et al., 2014; Queiroz et al., 2017). Different types of berries have been reported to exert neuroprotective effects via modulating enzymes with antioxidant activity, improving cognitive function, decreasing lipid peroxidation and ROS production and increasing SOD expression in an *in vitro* model of glutamate-induced toxicity as well as *in vivo* models of neurodegenerative diseases and senescence-accelerated rats (Kolosova et al., 2006; Forbes-Hernandez et al., 2016; Lee et al., 2017). For instance, addition of pomegranate antioxidant extracts to the diet of obese rats exerted antioxidant effects and reversed hyperlipidemia and cerebral oxidative stress (Amri et al., 2017; Oviedo-Solís et al., 2017). Also, phenols were shown to exert neuroprotective effects in neuroblastoma cells and HT22 hippocampal mouse cells instigated with oxidative stress (Tavares et al., 2013; Vepsäläinen et al., 2013).

In the present study, we explored whether different extracts isolated from *P. peruviana* could counteract the oxidative damage induced by rotenone in T98G cells. Our findings showed that EF and AD extracts improved cell survival and preserved mitochondrial functions in rotenone-treated cells.

*P. peruviana* has been reported to exert beneficial effects in various chronic diseases. This plant contains peruvioses and sucrose esters that are responsible for the reported hypoglycemic and hypocholesterolemic effects and the observed benefits reported in improving health conditions like cancer, cardiovascular and neurodegenerative diseases (Ramdan, 2011; Abdel Moneim et al., 2014; Al-Olayan et al., 2014; Bernal et al., 2018). Other protective actions of *P*. *peruviana* rely on its antioxidant, anti-inflammatory and anti-apoptotic properties that are probably exerted by the bioactive compounds present in leaves, stems, fruits and calyx of the plant (Martínez et al., 2010; Franco et al., 2014; Toro et al., 2014). Indeed, previous studies have shown a high content of polyphenols and carotenoids that are strong ROS scavengers, in the plant fruits (Lan et al., 2009; Yen et al., 2010; Ramdan, 2011; Dkhil et al., 2014; Toro et al., 2014; Yildiz et al., 2015). It is quite known the presence of these bioactive compounds in different parts of *P*. *peruviana*. However, in our study, the analysis of phenols has been limited to calculated equivalents of gallic acid with spectrophotometric or HPLC methods (Hassanien, 2011; Zhang et al., 2013; Cortés Díaza et al., 2015).

Exposure of astrocytic cells to rotenone is a validated method to simulate the neuropathological conditions of PD (Cabezas et al., 2015) and allows preliminary screening of neuroprotective compounds with antioxidant properties. This experimental approach allowed us to determine which fractions of the *P. peruviana* whole extract have antioxidant or cytotoxic effects. This information will be instrumental to identify beneficial secondary metabolites from *P. peruviana*, which might be tested in future *in vivo* models to validate any therapeutic effects. To assess the beneficial actions of the phytochemicals from *P. peruviana*, further analytical studies should be performed using mass spectrometry in order to better understand their interference and modulatory effects on the intrinsic pathological mechanisms of these diseases.

Many beneficial properties of *P. peruviana* pure extracts had already been identified but our work is the first to evaluate the effects of *P. peruviana* with different polarities in an *in vitro* model of a neurodegenerative disease that is associated with oxidative stress. Our results indicated that the effect of the extracts on cell viability was dependent on the concentration and incubation time applied on the T98G cells. Low and medium polarity extracts, which are those obtained by petroleum benzene and dichloromethane, showed a significant cytotoxic effect that was dependent on the time and the concentration applied (Supplementary Figure 1). On the other hand, extracts of medium-high polarity and polar extracts showed lower cytotoxicity (Supplementary Figure 2). In fact, when the AD, AF, EF, and ED extracts were tested, minor increases in cell viability were observed. For instance, cells exposed to AD extract at 25 μ g/ml for 12 h showed a 53% increase in cell viability (Supplementary Figure 2). The cytotoxic effect of low polarity extracts is consistent with the findings of previous studies; for instance, benzene and dichloromethane extracts from *Ficus Sycomorus* were found to be less toxic compared with those obtained with ethyl acetate or ethanol (Al-Matani et al., 2015; Tang et al., 2015). Consistent with previous studies, extracts of goldenberry obtained with ethanol and polar solvents (acetone and ethyl acetate) showed higher biological activities and neuroprotective effects.

Previous studies reported an increase in cell survival after exposure to 1-methyl-4-phenyl-1,2,3,6-tetrahydropyridine (MTPT)-treated cells to goldenberry extracts. This increase in viability was accompanied by cytoplasmic membrane protection from ROS damage, increased expression of the antioxidant enzymes SOD and catalase, as well as improved cognitive function and memory (Faria et al., 2005; Fortalezas et al., 2010; Debnath et al., 2011; Jeong et al., 2013). Also, in *in vitro* and *in vivo* studies performed by Kim et al. (2010), a decrease in symptoms related to PD such as bradykinesia and loss of dopaminergic neurons was noted after exposure to ethanolic extract of blackberries. The authors postulated that the mulberry extract and its polyphenols could serve as natural drugs for the prevention and treatment of PD (Kim et al., 2010).

It should be noted the overall phenolic content of the most polar fractions did not correlate with the percentage of cell survival. Although the Folin-Ciocalteu reagent assay indicates the total phenols of a sample, there are differences in the methods used to obtain the extracts. The presence of water changes the elution capacity of solvents. Thus, in the fresh fruit extract, water facilitates the elution of some hydrosoluble components of the fruit into ethanol. Ethanol was the first fraction obtained, making it possible to elute the more significant portion of the polar elements associated with water. On the other side, with the dehydrated fruit, the solvent elution capacity varied and the more polar components are extracted by acetone and are eluted with higher selectivity than the less polar components. Among the phenols, the most polar compounds are the glycosylated flavonoids. These extraction differences and variations in antioxidant compounds may somehow explain the increase in the percentage of cell survival, mainly observed with EF and AD extracts. Furthermore, the Folin-Ciocalteu reagent assay is not specific for phenolic components, as it also recognizes the phenolic structures of proteins and many other (reducing) compounds (Huang et al., 2005; Prior et al., 2005). In our study, to minimize the content of protein-related phenolic structures, the cellular components were discarded by centrifugation. Further studies should determine and characterize the phenolic components of *P. peruviana*, an analysis that goes beyond the goal of our present study.

Enhanced production of ROS in the brain is a phenomenon that occurs in a large number of neurodegenerative diseases (Vauzour et al., 2008), suggesting a role for oxidative stress in triggering cell death. Decrease in superoxide radical and hydrogen peroxide levels observed in cells treated simultaneously with rotenone and EF or AD suggests that these extracts possess antioxidant activity (Figures 3A,F). It is not known whether the effect elicited by these extracts is due to free radical scavenging or reduction of ROS formation. Our findings, however, show that these extracts can directly reduce oxidative stress (Figures 2C,D,E). This is supported by the reduction in membrane lipid oxidation (3% decrease in HNE fluorescence), which may be associated with a reduction of intracellular ROS levels. In this regard, high concentrations of carotenoids, the pigments that impart the yellow color of the fruit, may play a fundamental role in the scavenging of oxidant compounds, since these molecules have a high affinity for ROS (Rodriguez-Amaya, 2010). Likewise, the expression of antioxidant enzymes may be induced by an increase in ROS that activates the cellular defense system, which can either inhibit oxidative damage or trigger an apoptotic process in astrocytes (Barreto G. E. et al., 2011; Barreto G. et al., 2011). An increase in ERK expression could activate cell survival pathways and reduce cell death (Essa et al., 2012). Nitric oxide is a reactive nitrogen species and can further promote oxidative damage (Chaturvedi and Flint Beal, 2013). We found a slight increase in nitrite concentration in cells treated with rotenone and a decrease when the cells were treated simultaneously with the extracts but the variations were not significant compared to the control.

The mitochondria play a fundamental role in the regulation of cellular oxidative status as well as response to oxidative stress (Federico et al., 2012). For this reason, mitochondrial dysfunction is directly associated with increased ROS content in the brain in most neurodegenerative diseases (Cabezas et al., 2012; Chaturvedi and Flint Beal, 2013; Ruszkiewicz and Albrecht, 2015). In fact, mitochondrial dysfunction has been established as one of the early and vital features of neurodegeneration (Federico et al., 2012). The results presented in this study indicated a decrease in the mitochondrial membrane potential in cells exposed to rotenone. The loss of mitochondrial membrane potential is one of the initial steps that occur in the process of mitochondrial dysfunction and subsequent cell death (Federico et al., 2012). This phenomenon may also explain our observation of decreased cell viability in rotenone-treated cells. Nevertheless, the results showed that EF and AD extracts preserved mitochondrial membrane potential against rotenone stimulation (Figure 3A), suggesting that these extracts are mitochondrial protectors.

Another indicator of mitochondrial dysfunction is alteration of mitochondrial mass (Ávila Rodriguez et al., 2014; Cabezas et al., 2015). An increase in mitochondrial mass was detected in cells treated with rotenone, with an opposite turnout when cells were treated with either EF or AD. We postulate that the slight increase in mitochondrial mass in the rotenone group may represent a process of abnormal mitochondrial dynamics due to the loss of fusion/fission mitochondrial homeostasis (Parrado-Fernández et al., 2016). In fact, there are reports in the literature indicating that cellular stress promotes the mitochondrial fusion process (Westermann, 2010), which may explain the increase of mitochondrial mass in the cells exposed to rotenone. In this sense, it is reasonable to suggest that the decrease in the mitochondrial mass induced by the exposure of cells to the extracts was due to the ability of the extracts in reducing oxidative stress and maintaining mitochondrial membrane potential. However, future studies investigating the mode of action of phytochemicals in modulating mitochondrial dynamics are necessary to clarify the mechanisms underlying the effect of these extracts on the mitochondrial mass. On the other hand, a decrease in mitochondrial Ca^2+^ in the rotenone-treated cells was also observed in our study, yet these levels slightly increased when the cells were co-treated with AD (Figure 3C). These findings are in agreement with previous studies showing that cranberries prevent deregulation of the Ca^2+^ in hippocampal cells in rats (Brewer et al., 2010). However, continuous measurements to test the effects of extracts on rapid variations of calcium ion in the RE and mitochondria of astrocytes (Abeti and Abramov, 2015) are still required.

The antioxidant capacity of a compound can be extrapolated to activities that go beyond capturing or preventing the formation of ROS (Nones et al., 2010). For example, it has been stated that the antioxidant capacity of a compound is greater when it also stimulates antioxidant-related pathways such as ERK signaling (Spencer, 2008). In this direction, it has been reported that flavonoids have strong antioxidant activities owing to their capacity in stimulating survival pathways involving enzymes such as ERK1 and 2. In our study, increased expression of ERK1 protein was observed when the cells were co-treated with rotenone and EF. Indeed, cells treated with AD extract showed higher expression of SOD and lower concentration of superoxide radical. These findings suggest that the tested extracts might enhance the endogenous antioxidant defense system (Halliwell, 2007). Nonetheless, it would be necessary to identify the active ingredients in these extracts. In future experiments, more refined analysis of subfractions of the tested extracts using HPLC and Mass spectrometry techniques are needed to elucidate the identity and quantity of the bioactive compounds. Finally, verification of the observed antioxidant effects of *P. peruviana* extracts in animal models of neurodegenerative diseases associated with oxidative stress merits further investigation.

## Conclusions

The present study suggested that the EF and AD extracts from *P. peruviana* fruits may have cytoprotective and antioxidant effects in brain cells exposed to neurotoxic stimuli. These effects were expressed as reduced cell death, preserved mitochondrial functions and attenuated nuclei damage. Moreover, our results demonstrated that: (i) different effects of *P. peruviana* fruit extracts depend on the polarity of the extraction solvent; (ii) extracts of medium-high polarity (namely AD and EF) have protective effects on the mitochondria; and (iii) AD and EF extracts might induce cytoprotective actions in rotenone-treated cells by enhancing the cellular antioxidant content which has a positive impact on cell survival. More studies should be performed with the aim of assessing the bioavailability and bioactivity of *P. peruviana* extracts *in vivo*. Finally, our study opens a new avenue of research aiming at the identification of natural compounds as potential drugs to treat neurodegenerative diseases.

## Author contributions

NA-M, JR, JZ-R, LG, BB-B, VE, GA, AS, GMA, and GB designed the experiments. NA-M, GB, GA, AS, GMA, VE, JR, BB-B analyzed the results. NA-M, GA, AS, GMA, VE, BB-B, GMA and GB wrote and revised the manuscript. All authors have approved the final revised manuscript.

### Conflict of interest statement

The authors declare that the research was conducted in the absence of any commercial or financial relationships that could be construed as a potential conflict of interest.
