# Supplementary material for: Extracts of Physalis peruviana Protect Astrocytic Cells Under Oxidative Stress With Rotenone
Source: Front Chem. 2018 Jul 20;6:276. doi: 10.3389/fchem.2018.00276 (PMC6108337; doi:10.3389/fchem.2018.00276)
Supplement: Supplementary file 1 [file Presentation_1.PDF]

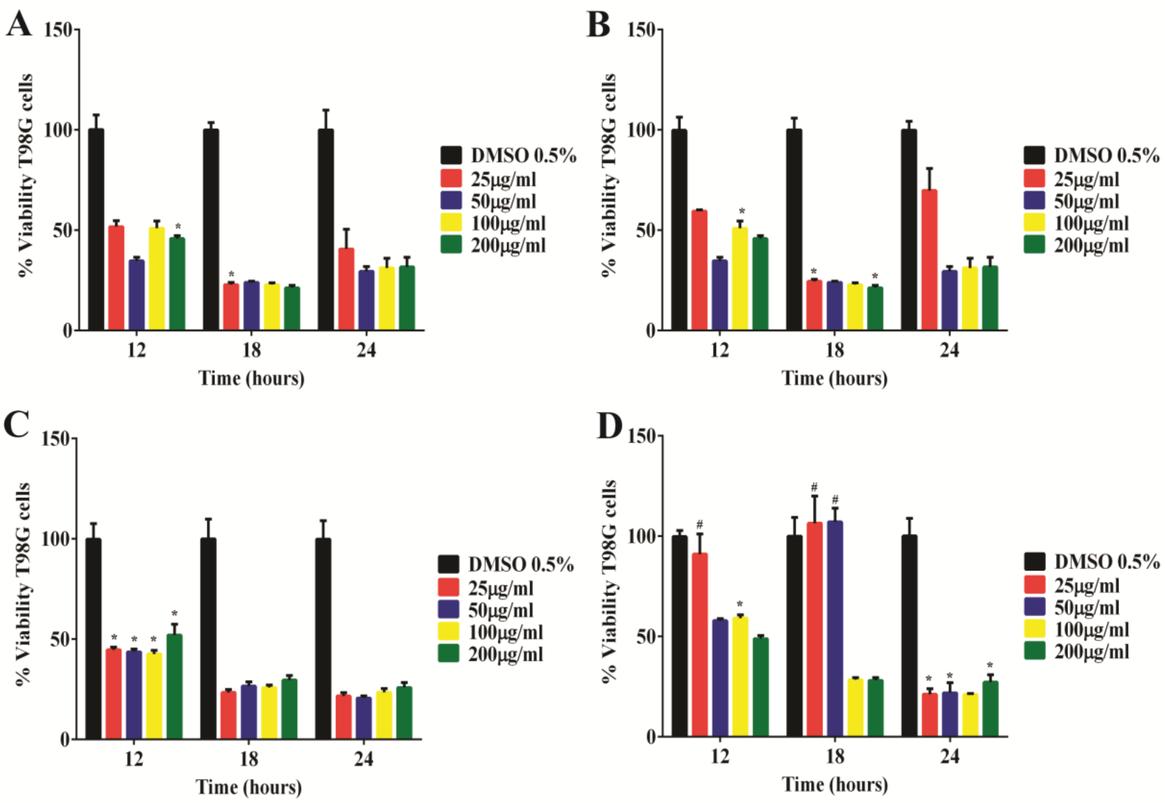

Figure 1. Effect of extracts from *Physalis peruviana* fruit on cell viability in T98G cells at 12, 18 and 24 hours of exposure by MTT test. The extracts analyzed had a high cytotoxic effect. A) Petroleum benzene extract of dehydrated fruit, B) Dichloromethane extract of dehydrated fruit. C) Petroleum benzene extract of fresh fruit and D) Dichloromethane extract of fresh fruit. # Significant differences compared to control (0.5 % DMSO) ($p<0.0001$). * Significant differences ($p<0.02$) between different times.

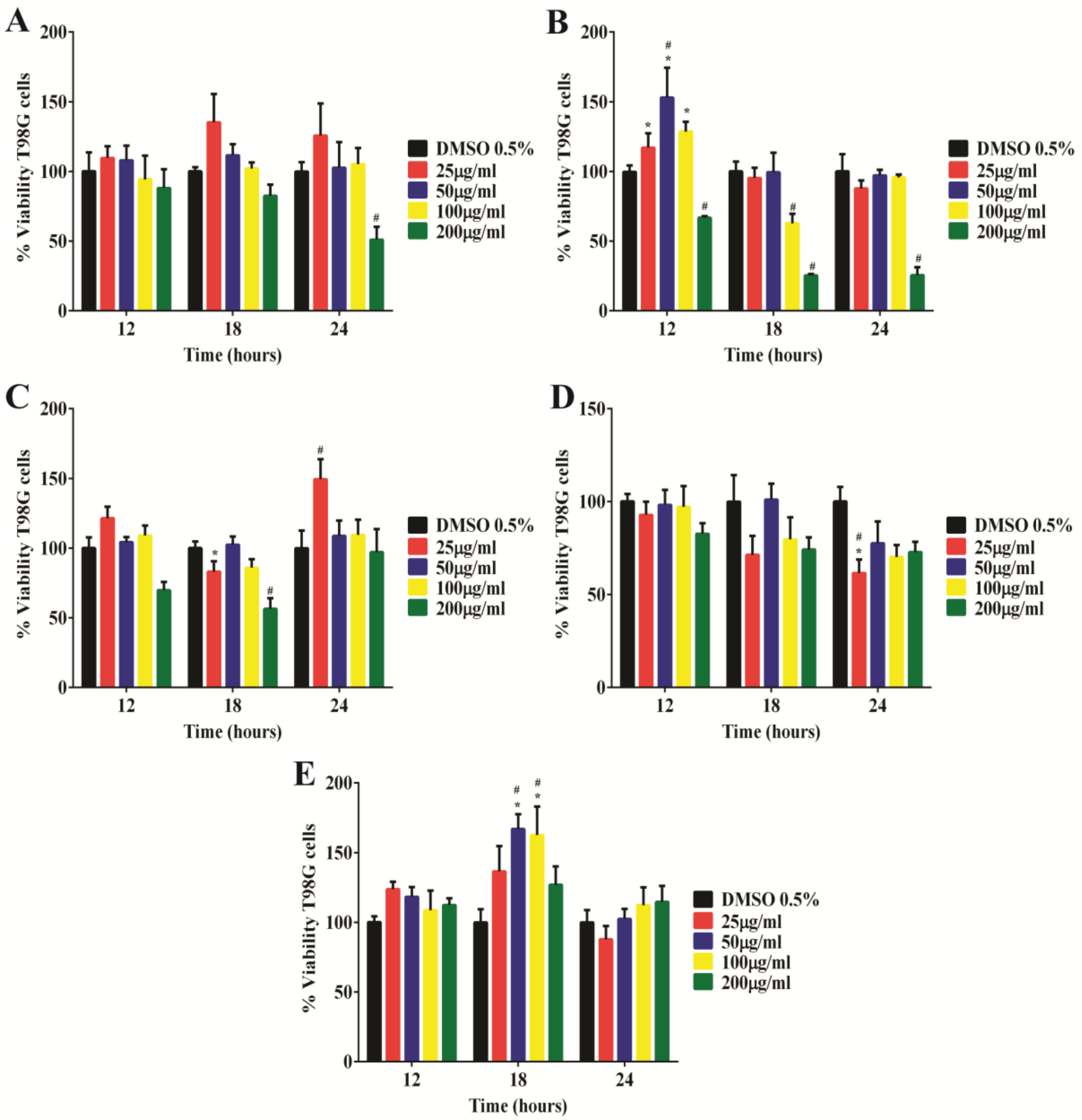

Figure 2. Effect of extracts from *Physalis peruviana* fruit on cell viability in T98G cells at 12, 18 and 24 hours of exposure by MTT. The extracts had a low cytotoxic effect. A) Ethanolic extract of dehydrated fruit, B) Acetone extract of dehydrated fruit, C) Ethanolic extract of fresh fruit and D) Ethyl acetate extract of fresh fruit. E) Lyophilized extract. # Significant differences compared to control (0.5 % DMSO) ($p<0.0001$). * Significant differences between the different times ($p< 0.03$).
